# Alternative 3′UTR expression induced by T cell activation is regulated in a temporal and signal dependent manner

**DOI:** 10.1038/s41598-024-61951-1

**Published:** 2024-05-14

**Authors:** Davia Blake, Matthew R. Gazzara, Isabel Breuer, Max Ferretti, Kristen W. Lynch

**Affiliations:** 1 Department of Biochemistry and Biophysics, Perelman School of Medicine, University of Pennsylvania, Philadelphia, PA 19104 USA; 2 Immunology Graduate Group, Perelman School of Medicine, University of Pennsylvania, Philadelphia, PA 19104 USA; 3 Genomic and Computational Biology Graduate Group, Perelman School of Medicine, University of Pennsylvania, Philadelphia, PA 19104 USA; 4 Genetics and Epigenetics Graduate Group, Perelman School of Medicine, University of Pennsylvania, Philadelphia, PA 19104 USA

**Keywords:** Alternative polyadenylation, 3′UTR, CD3, CD28, T cell receptors, RNA, Adaptive immunity

## Abstract

The length of 3′ untranslated regions (3′UTR) is highly regulated during many transitions in cell state, including T cell activation, through the process of alternative polyadenylation (APA). However, the regulatory mechanisms and functional consequences of APA remain largely unexplored. Here we present a detailed analysis of the temporal and condition-specific regulation of APA following activation of primary human CD4^+^ T cells. We find that global APA changes are regulated temporally and CD28 costimulatory signals enhance a subset of these changes. Most APA changes upon T cell activation involve 3′UTR shortening, although a set of genes enriched for function in the mTOR pathway exhibit 3′UTR lengthening. While upregulation of the core polyadenylation machinery likely induces 3′UTR shortening following prolonged T cell stimulation; a significant program of APA changes occur prior to cellular proliferation or upregulation of the APA machinery. Motif analysis suggests that at least a subset of these early changes in APA are driven by upregulation of RBM3, an RNA-binding protein which competes with the APA machinery for binding. Together this work expands our understanding of the impact and mechanisms of APA in response to T cell activation and suggests new mechanisms by which APA may be regulated.

## Introduction

Protein expression and identity is regulated in cells not only by transcription by also by myriad steps of processing of a nascent pre-mRNA transcript. Such pre-mRNA processing includes the splicing out of introns to generate an open reading frame, as well as 3′ end processing (aka polyadenylation) that serves to terminate transcription and mark the 3′ end of the transcript for export and translation through the addition of a tail of 100–200 adenosines^1^. The location along the pre-mRNA at which the transcript is cleaved for addition of the adenosine tail also sets the identity of the 3′ untranslated region (3′UTR) of the message. Given that the 3′UTR typically encodes information that determines the stability, translation efficiency or localization of a message^2–5^, the location of cleavage and polyadenylation is important for accurate protein expression even if it doesn’t directly impact the coding sequence of the mRNA.

Importantly, the location of cleavage and polyadenylation is dynamically regulated for many transcripts^5,6^. Indeed, it is now well documented that alternative use of competing sites of cleavage and polyadenylation within a given transcript, by a process termed alternative polyadenylation or APA, occurs in more than half of protein coding genes and exhibits tissue- and condition-specific regulation^6–8^. APA impacts the length of the final mRNA, with the potential to truncate the open reading frame and thus the encoded protein, or alter the presence of regulatory elements in the 3′UTR to control protein expression, localization or even biologic activity^5,6,9,10^.

Previous studies have demonstrated widespread APA upon T cell activation, leading to robust global shortening of 3′UTRs^11,12^. Interestingly, pervasive 3′UTR shortening is a common phenomenon seen in proliferative cells, such as cancer cells, compared to more differentiated cells^11^. Conversely, highly differentiated cell types, such as neurons, exhibit global 3′UTR lengthening compared to T cells^13^. However, it remains unclear what the overall biological significance is of a global shortening or lengthening for determining cellular activity.

As more examples of APA have been studied in detail, there has also begun to be a greater understanding of the mechanisms driving APA. Cleavage and polyadenylation is accomplished by a large multicomponent complex that recognizes a series of sequences elements that define polyadenylation (PAS) sites^6,14^. The core PAS element is typically an AAUAAA hexanucleotide that is bound by the Cleavage and Polyadenylation Specificity Factor (CPSF) complex. The interaction of CPSF with the hexanucleotide is further enhanced by the Cleavage Stimulatory Factor (CstF) complex bound to GU-rich sequences downstream of the AAUAAA, and the Cleavage Factor I (CFI) dimer bound to a UGUA motif upstream of the AAUAAA^14,15^. All of these sequence elements exist with some degeneracy, such that the use of competing PAS sites is typically dictated by the inherent “strength” of the sequence elements (i.e. similarity to concensus) and the expression of the protein factors which comprise the CPSF, CstF or CFI complexes. For example, the increased expression of CstF-64 within proliferative cells has been shown to be one mechanism to promote global 3′UTR shortening, due to increased likelihood of CstF-64 binding to weak proximal PAS sequences as its concentration increases^16^. As an alternative model of regulation, competition between general RNA binding proteins (RBPs) and the polyadenylation machinery has also been shown to regulate PAS choice. Indeed, we have shown that the RBP CELF2 competes with CFIm and CstF-64 for binding to UGU-rich sequences, thereby reducing use of PAS sites surrounded by strong CELF2 binding sites under condition of increased CELF2 expression^17^.

Despite the fact the T cell activation was one of the first examples of cellular proliferation shown to correlate with widespread APA^11^, there remains minimal investigation of the target genes, functional impact or mechanistic drivers of APA downstream of T cell signaling. Recently we carried out deep sequencing of RNAs expressed in naïve primary human T cells compared to those stimulated in vitro through the T cell receptor (CD3) and/or the co-stimulatory receptor CD28^18^. Co-stimulation of CD28 together with CD3 promotes cell survival^19,20^. By analyzing alternative splicing in naïve and stimulated human T cells, we found that CD28 co-stimulation increased the alternative splicing of several apoptotic regulators to promote an anti-apoptotic response^18^.

Here we analyze our T cell activation RNA-Seq dataset for alternative polyadenylation to better elucidate the regulation of global APA changes in T cells. We find that global APA changes are regulated temporally and are sensitive to CD28 costimulatory signaling, in a manner similar to splicing and differential expression changes. Consistent with previous smaller-scale studies^11^, we find that genes which undergo APA upon T cell activation largely exhibit 3′UTR shortening. While this shortening does not correlate with any uniform trends in the steady-state abundance of the corresponding mRNA or encoded protein, we do identify many individual cases of genes encoding key regulators of T cell effector function that exhibit changes in both APA and mRNA abundance. Moreover, genes that undergo 3′UTR shortening in activated T cells are enriched for those that are localized to TIS granules, cytoplasmic compartments of translation which have been shown to influence protein–protein interactions^21^. Finally, we find that while upregulation of the core polyadenylation machinery likely controls APA following prolonged T cell stimulation; a significant set of APA changes occur prior to cellular proliferation or changes in the abundance of polyadenylation factors. We provide evidence suggesting that at least a subset of these early changes in APA are driven by upregulation of the RBP RBM3, which we propose binds around distal PAS hexamers to promote use of more proximal PAS sites. Together this work expands our understanding of the impact and mechanisms of APA in response to T cell activation.

## Results

### Global polyadenylation upon T cell activation leads to widespread 3′UTR shortening in a temporal and signal-specific manner

**Figure 1:**
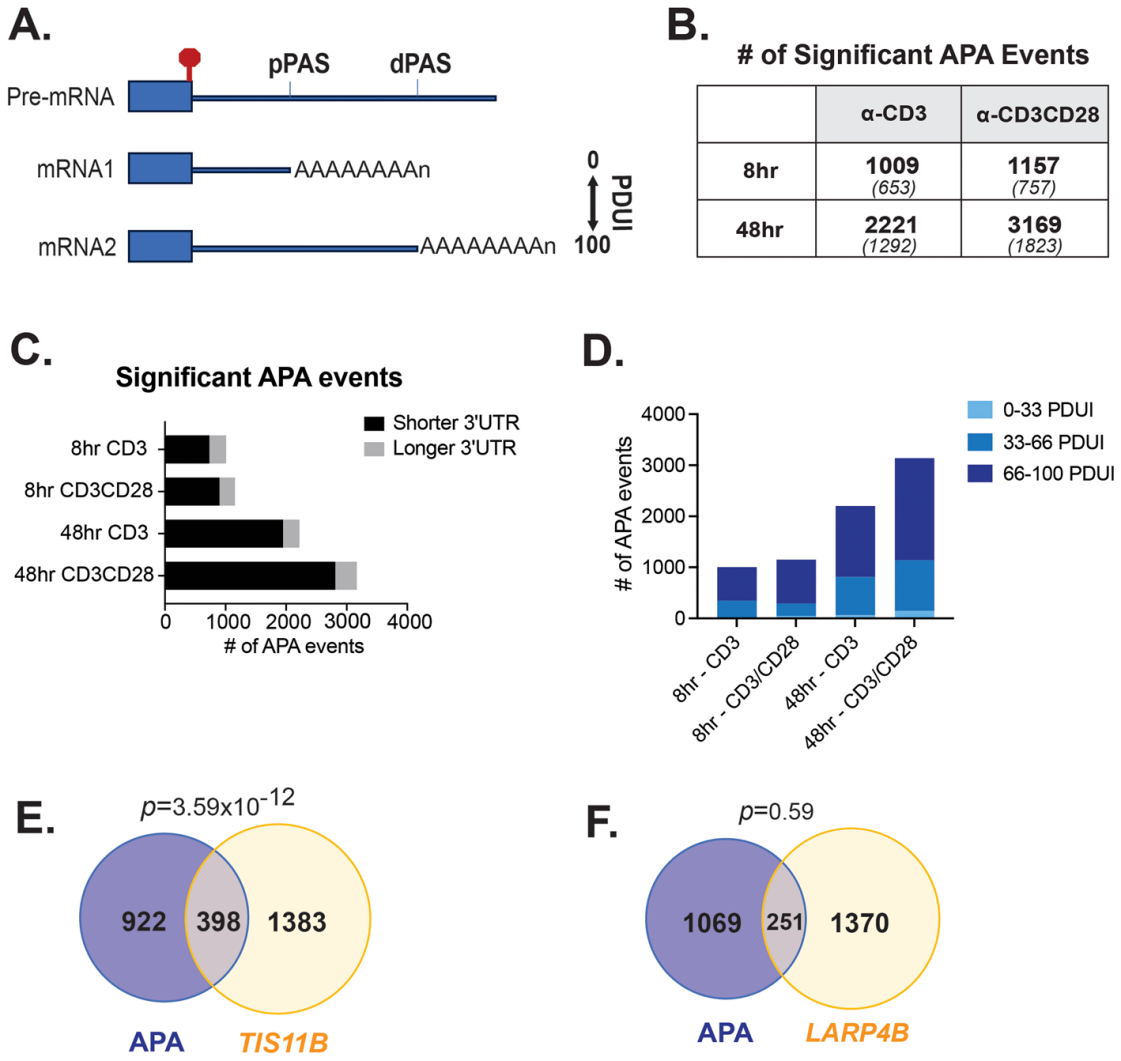
APA is regulated in a temporal and condition-specific manner in primary human T cells. (**A**) Schematic of regulation of 3′UTR length by APA. Box is last coding exon, stop sign is translation stop codon and thick blue line is 3′UTR. Proximal (pPAS) and distal (dPAS) sites of cleavage and polyadenylation are noted. Use of pPAS results in mRNA1 and corresponds to a PDUI of 0, while use of dPAS leads to mRNA2 and a PDUI of 100. (**B**) Bold number indicates number of APA events that exhibit significant (> 20% dPDUI, < 0.05 p-value) change under indicated conditions of treatment of primary human naïve CD4^+^ T cells. Number in parentheses indicates number of genes impacted. Since some genes have more than one pairwise APA change the number of genes is less than the number of events. (**C**) Plot of APA events from panel (**B**) with number of events that lead to 3′UTR shortening or lengthening indicated. (**D**) Plot of APA events from panel (**B**) with PDUI in naïve cells indicated. (**E**) Overlap of genes that exhibit 3′UTR shortening 48 h after CD3/CD28 costimulation with those bound by TIS11B. (**F**) Overlap of genes that exhibit 3′UTR shortening 48 h after CD3/CD28 costimulation with those bound by LARP4.

Previous studies have demonstrated global 3′UTR shortening upon T cell activation^11^. We sought here to further elucidate the regulation of APA that leads to such shortening, and to discover biological consequences of such APA changes in primary human CD4^+^ T cells. To this end, we reprocessed previously published RNA-Seq data from naive (CD45R0−) CD4^+^ primary T cells from 3 healthy human donors stimulated ex vivo with anti-CD28, anti-CD3, or anti-CD3/CD28 for 8 and 48 h (GSE135118)^18^ using the DaPars algorithm^22,23^. The robustness of the T cell stimulation and the reproducibility of the effect of CD28 costimulation between these human donors was previously confirmed by the expression of CD69 and IL2 respectively^18^. DaPars provides de novo identification of APA at the transcript level at single nucleotide resolution. APA events within a given gene are quantified as Percentage of Distal polyA Site Usage Index (PDUI), which is simply a measure of the percentage of transcripts using the more distal (dPAS) relative to the proximal PAS (pPAS). By definition, favored use of the dPAS, leading to a longer 3′UTR, yields a PDUI closer to 100, while a shorter 3′UTR is closer to 0 PDUI (Fig. 1A). Significant changes in APA are defined as genes for which the difference in the average PDUI (delta PDUI or dPDUI) between stimulated and unstimulated conditions has an absolute value greater than 20 and an adjusted p-value < 0.05. This is a common threshold for DaPars that we and others have found allows for reproducibility and validation of robust, biologically meaningful APA changes^17^.

As quantified in Fig. 1B, at 8 h of stimulation by anti-CD3, or costimulated with both anti-CD3 and anti-CD28 (anti-CD3/CD28), we observe 1009 and 1157 significant APA changes occur, respectively. Upon 48 h of stimulation this number increases to 2221 and 3169 APA events induced by anti-CD3 or anti-CD3/CD28, respectively. Note that since stimulation with anti-CD28 alone is not a biologically meaningful condition and has yielded highly noisy and variable results in our studies of alternative splicing^18^, we did not assess this condition in the present study. Since previous studies have indicated that robust global shortening occurs upon T cell activation^11^, we asked if the proportion of APA events that present 3′UTR shortening or lengthening changes temporally or is specific to T cell stimuli. Consistent with published reports of cell proliferation^8,11^, we find that the majority of the APA events induced upon stimulation of primary T cells result in shortening of the 3′UTR (i.e. increased use of proximal APA site) (Fig. 1C). While the sheer number of significant APA events increases between early and late T cell activation, the dominance of 3′UTR shortening versus lengthening does not significantly change between early or late T cell activation (Fig. 1C). In addition, the proportion of events that display 3′UTR shortening is not dependent on CD28 costimulation (Fig. 1C). Consistent with this global shortening, analysis of the basal, unstimulated PDUI levels for genes that exhibit APA upon stimulation reveals that these transcripts predominantly express long 3′UTRs (PDUI > 66) in unstimulated cells (Fig. 1D). Overall, this analysis demonstrates a strong bias toward longer 3′UTRs in unstimulated T cells with a global 3′UTR shortening in response to activation across all times and stimuli conditions tested.

### Genes that undergo APA upon T cell activation are enriched for association with TIS11B and encode functions critical for immune responses

To investigate the biological significance of APA events that exhibit differential 3′UTR shortening or lengthening upon stimulation, we first utilized gene ontology analysis to determine if there are unique enrichment of biological functions. The few genes that exhibit longer 3′UTRs upon 8 h of CD3 and CD3/CD28 activation do not present any notable enrichments of biological processes (Fold Enrichment > 2), however by 48 h there is clear enrichment of genes related to TOR signaling amongst those with lengthened 3′UTRs (Supplemental Fig. S1). By contrast, genes that present shorter 3′UTRs upon 8 h of CD3 stimulation are enriched for processes that include Golgi vesicle transport, vesicle organization and RNA processing, while genes with shortened UTRs induced by CD3/CD28 co-stimulation are enriched for RNA localization and phospholipid biosynthetic processes (Supplemental Fig. S1). After 48 h of stimulation with anti-CD3, genes that exhibit shorter 3′UTRs are themselves associated with RNA processing, especially polyadenylation; while genes which have shorter 3′UTRs following stimulation with both anti-CD3 and CD28 are associated with mitochondrial function. Of note, while UTR length can significantly impact the expression of individual genes or proteins, we and others find little global correlation between UTR length and gene or protein expression^12,17,24^ (Supplemental Figs. S2, S3). Therefore, we cannot make general statements about increasing or decreasing activity of any specific functional pathway, although we consider it likely that the changes in UTR length in most or all of these genes cause alterations in protein expression on a case-by-case basis (see below).

Recently, it has been shown that 3′UTR identity also controls spatial localization of translation^21,25^. In particular, interaction of mRNAs with the protein TIS11B localizes translation within phase-separated ER-proximal TIS-granules. TIS-granules serve to concentrate particular mRNAs and promote co-translational protein–protein interactions and assembly of protein complexes^21,26^. Interestingly, we find that genes that exhibit 3′UTR shortening in our dataset are significantly enriched for genes that are bound by TIS11B^27^ (Fig. 1E). By contrast, there is no enrichment of genes with activation-regulated APA for binding to LARP4B (Fig. 1F), which marks mRNAs for localization to the diffuse cytosol^27^. This data suggests that the functional impact of 3′UTR shortening in T cells may include TIS11B-dependent regulation of protein interactions even in the absence of changes in protein or gene abundance.

**Figure 2:**
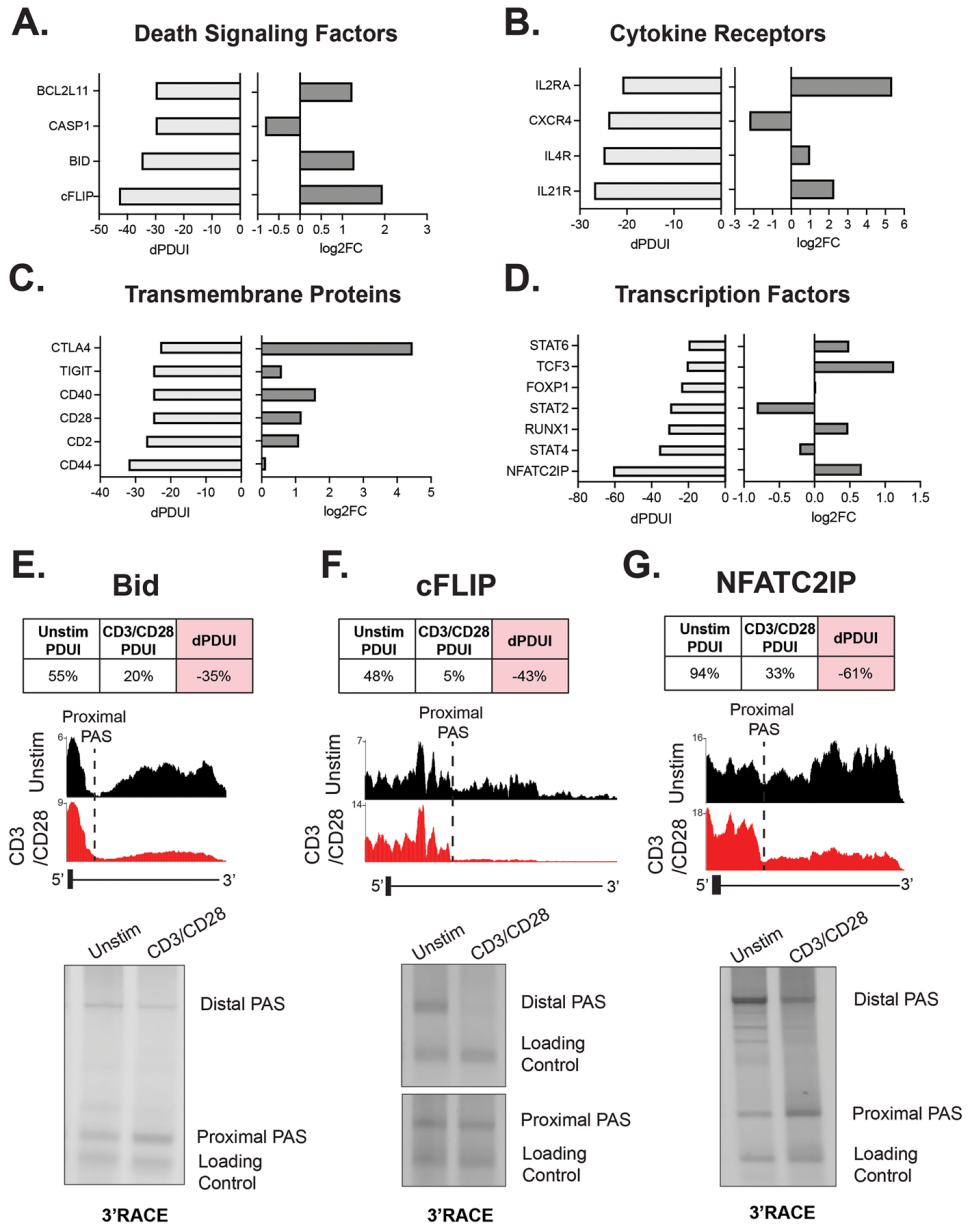
Characterization of immune-relevant APA events. (**A**–**D**) Immune-relevant genes that comprise of death signaling factors, cytokine receptors, transmembrane proteins, and transcription factors that undergo significant APA changes upon 48 h-CD3/CD28 stimulation. Bar graphs display dPDUI (48 h CD3/CD28—Unstim) levels of APA changes (left) and log2FC analyzed by RNA-seq (right). (**E**–**G**) Genome Browser tracks and 3′RACE experiments of examples of immune-relevant genes significantly regulated by APA changes. Tracks shown are the average reads across three donors. Y-axis represents reads per million sequences.

Although we don’t observe any enriched GO categories that are specifically related to T cell function (Supplemental Fig. S1), this does not preclude a potential role of APA in regulating specific genes with known T cell function. Therefore, we looked immune-relevant genes that exhibit altered APA within our above-described analysis (Fig. 2). Indeed at 48 h after CD3/CD28 co-stimulation we observe significant APA changes in multiple apoptosis factors (Bim, Bid, CASP1 and cFLIP), cytokine receptors (IL2RA, CXCR4, IL4R, and IL21R), transmembrane proteins known to exert influence on T cell activation (CTLA4, TIGIT, CD40, CD28, CD2, and CD44) and transcription factors that regulate immune function (STAT6, TCF3, Foxp1, STAT6, Runx1, STAT4, NFATC2IP) (Fig. 2A–D). In all of these above-mentioned genes, we observe shortening of the 3′UTR upon activation, as indicated by a negative dPDUI upon stimulation (dPDUI = PDUI stimulated-PDUI unstimulated). Moreover, for the majority of these genes, shortening of the 3′UTR is initially detected at 8 h, but does not reach significance until later time points following stimulation (Supplemental Fig. S4). Several of these 3′UTR shortening events, including in Bid, cFLIP and NFATC2IP, were validated using an orthogonal method of 3′RACE to assess PAS usage (Fig. 2E–G). Interestingly, many of the T cell related genes in which we observe APA exhibit increased mRNA and protein expression upon T cell stimulation, including genes critical for T cell function such as CTLA4 and IL2RA/CD25^28,29^. We have not yet investigated if APA in these mRNAs influences the ultimate expression. Nevertheless, the number of genes relevant to T cell activity that undergo APA certainly implies functional implications of this gene regulatory regime in tuning the T cell response to stimulation.

### CD28 costimulation enhances the extent of APA shortening for a subset of genes

Given the abundance and potential relevance of APA in activated T cells, we next sought to better understand the mechanistic drivers of APA following T cell stimulation. We initially focused on investigating the role of CD28 costimulation, as we observe different functional categories enriched between the CD3 alone and CD3/CD28 co-stimulation conditions (Supplemental Fig. S1) and CD28 costimulation enhances many aspects of T cell stimulation, including cell survival and genetic regulatory events such as gene expression changes and alternative splicing^30,31^. Indeed, we have recently shown that CD28 costimulation enhances a subset of alternative splicing changes to promote resistance to apoptosis^18^. However, a role for CD28 costimulation in the regulation of APA has not previously been reported.

**Figure 3:**
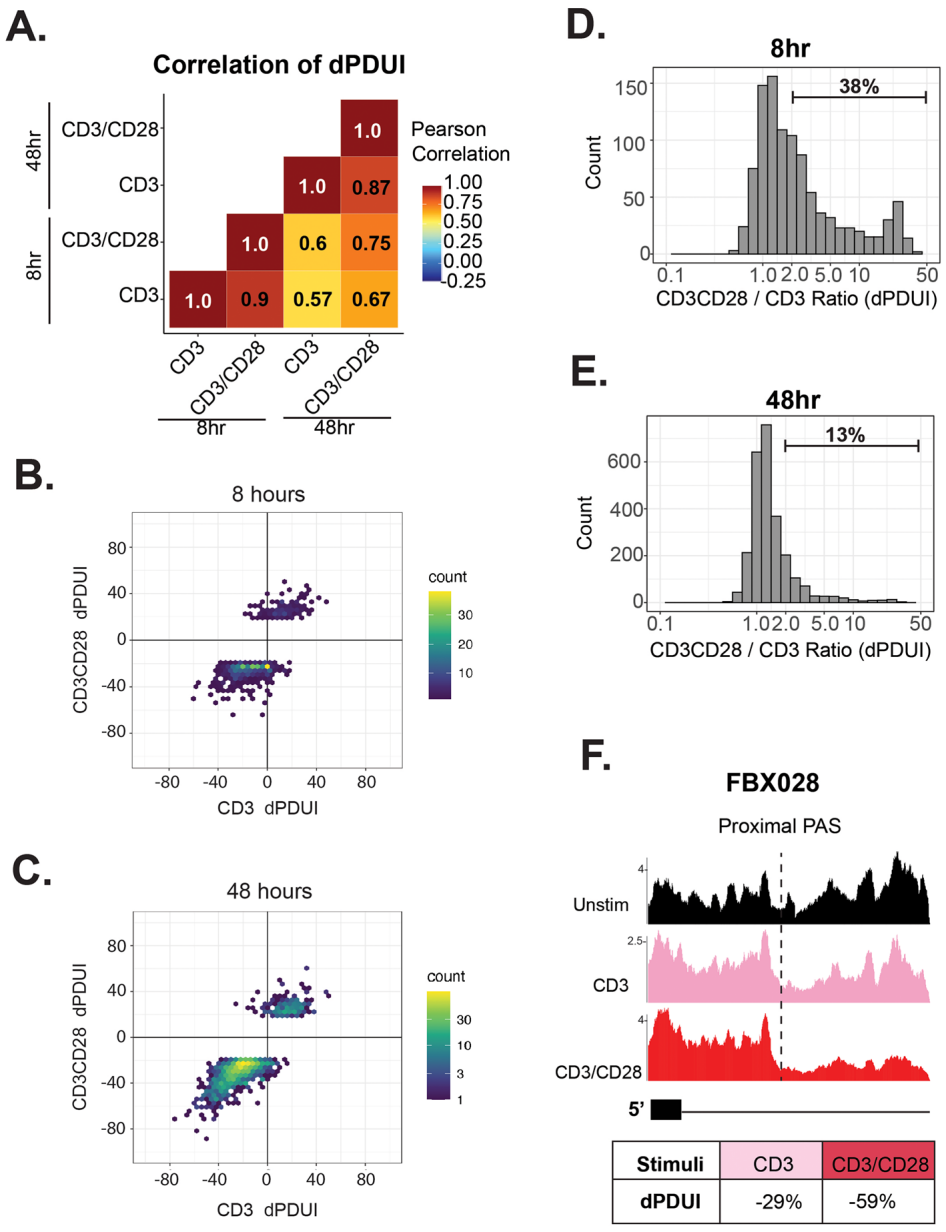
CD28 costimulation promotes magnitude of APA change for a subset of events. (**A**) Pairwise Pearson's correlation analysis of significant APA events. (**B**,**C**) Regression plot of the dPDUI of events that change at 8 (**B**) or 48 (**C**) hours under both stimulation with CD3 alone as well as CD3 and CD28 costimulation. (**D**,**E**) Ratio of dPDUI between CD3/CD28 costimulation and CD3 alone at 8 (**D**) and 48 (**E**) hours. Percent of events with a ratio greater than 2 is indicated. (**F**) An example of a genes that exhibits enhanced APA under conditions of CD28 costimulation. Tracks shown are the average reads across three donors. Y-axis represents reads per million sequences.

Although costimulation with CD28 results in more APA changes meeting the threshold for significance than are observed upon stimulation with CD3 alone (Fig. 1B–D), correlation of the APA changes induced by CD3 versus CD3/CD28 costimulation reveals linear regression lines close to 1 and Pearson's correlation scores > 0.87 (Fig. 3A–C). This trend between CD3 and CD3/CD28 mirrors global alternative splicing analysis^18^, and suggests that CD28 costimulation does not uniquely regulate a set of APA events but rather may enhance the extent of change in a subset of genes, as we have seen previously for alternative splicing^18^. To test this prediction, we assessed the dPDUI ratio between APA changes significantly induced by CD3/CD28 over that induced by CD3 alone (Fig. 3D,E). CD3 induced APA events with a very low dPDUI (< 1%) was set to 1% to prevent misleading skewing of the ratio quantification, as we and others have done in previous comparisons^18,32^. Consistent with our hypothesis, we find that after 8 h 38% of APA events exhibit a > 2-fold increase in extent change under conditions of CD28 costimulation versus CD3 stimulation alone (Fig. 3D), while at 48 h 13% of APA events exhibit such CD28 enhancement (Fig. 3E). These APA events regulated by CD28 costimulation present no significant enrichment of biological processes, but do include some dramatic differences such as observed in FBX028, a gene that encodes a ubiquitin ligase that regulates MYC transcription^33^, where significant 3′UTR shortening is only readily apparent in the context of additional CD28 costimulation (Fig. 3F).

We note that the events enhanced by CD28 costimulation at 8 h are largely distinct from those regulated at 48, and the majority do not exhibit any APA changes at the other time point (Supplemental Fig. S5). Thus, it does not appear that CD28 costimulation alters the time course of a signaling trajectory achieved by CD3 alone, but rather represents a unique regulatory program. Taken together, our data demonstrates that APA is sensitive to the presence of CD28 costimulation in a similar to manner of splicing changes^18^, however the mechanisms driving this CD28-dependent enhancement of APA, as for splicing, remains unknown (see “Discussion”).

### Temporal regulation of APA involves increased expression of RBM3 at 8 h and increased expression of the core polyadenylation machinery at 48 h

**Figure 4:**
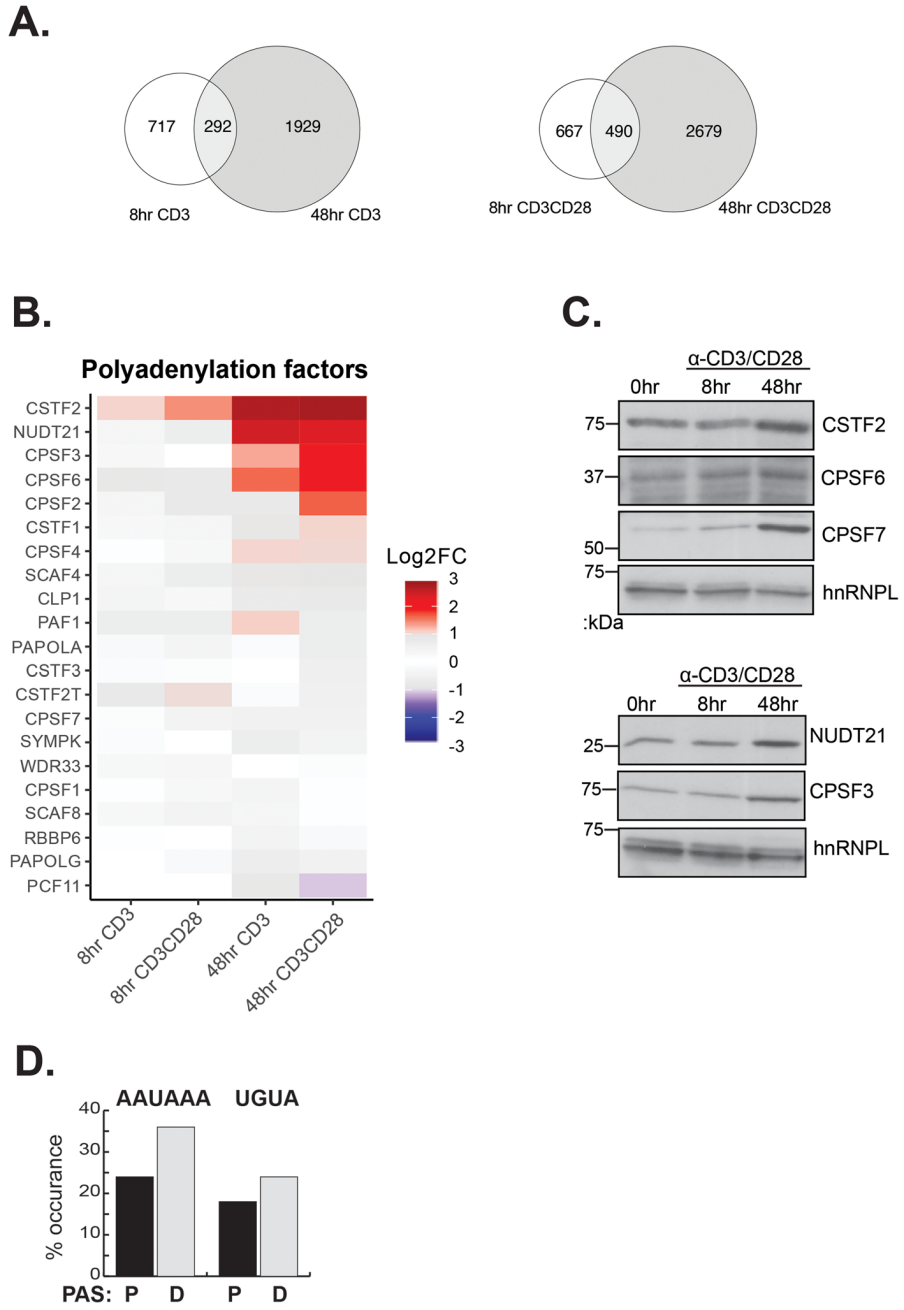
Temporal regulation of APA at 48 h correlates with an increase in APA core machinery. (**A**) Overlap of significant APA events between 8 and 48 h of T cell stimulation by stimulation condition. (**B**) Log2 fold changes of components of the core polyadenylation machinery as quantified by RNA-seq. (**C**) Western blot confirmation of protein increases at 48 h of CD3/CD28 costimulation for a subset of key polyadenylation machinery. (**D**) Occurrence of optimal polyadenylation signals at the pPAS and dPAS of genes that exhibit shortening at 48 h following costimulation.

We next turned our attention next to the temporal regulation of APA comparing events. Consistent with our GO analysis above (Supplemental Fig. S1) and the trajectory of APA of individual events upon CD28 costimulation (Supplemental Fig. S5), we find limited overlap of the genes that exhibit APA at 8 h post-stimulation versus those changing at 48 h (Fig. 4A), with a majority of genes undergoing APA uniquely at 48 h (Figs. 1B, 4A). Moreover, Pearson's correlation analysis suggests that APA changes induced between 8 and 48 h of T cell stimulation, by either stimuli, produces only moderate correlation rates (R^2^ = 0.57, R^2^ = 0.75; Fig. 3A).

The finding that distinct sets of APA events change at early and late time points suggests different mechanisms are at play to control polyadenylation at different times following stimulation. At 48 h post-stimulation cells are actively proliferating (Supplemental Fig. S6A), a feature that has been correlated with APA in previous studies^6,34^. Previous studies have also demonstrated that increased expression of core polyadenylation factors can drive 3′UTR shortening through increased occupancy and use of weak proximal (upstream) PAS elements that diverge from consensus^35^. Consistently, we find that mRNAs encoding several core components of the polyadenylation machinery (e.g. CSTF2, NUDT21, CPSF3, CPSF6, CPSF2, CSTF1, and CSTF4) exhibit a dramatic increase in expression at 48 h after stimulation with either CD3 or both CD3 and CD28 (Fig. 4B). For many of these we also confirmed increase expression of mRNA by qPCR (Supplemental Fig. S6B) and protein by Western blot (Fig. 4C). Of note, the proximal PAS sites that are enhanced 48 h after stimulation to shorten UTRs are less likely than their distal counterparts to have consensus PAS signals (Fig. 4D). Therefore, we predict that much of the APA change observed at 48 h is driven by the increase in polyadenylation machinery which leads to increased recognition of weak proximal PAS sequences, as has been observed upon other instances of 3′UTR shortening upon cell proliferation^34,36^.

**Figure 5:**
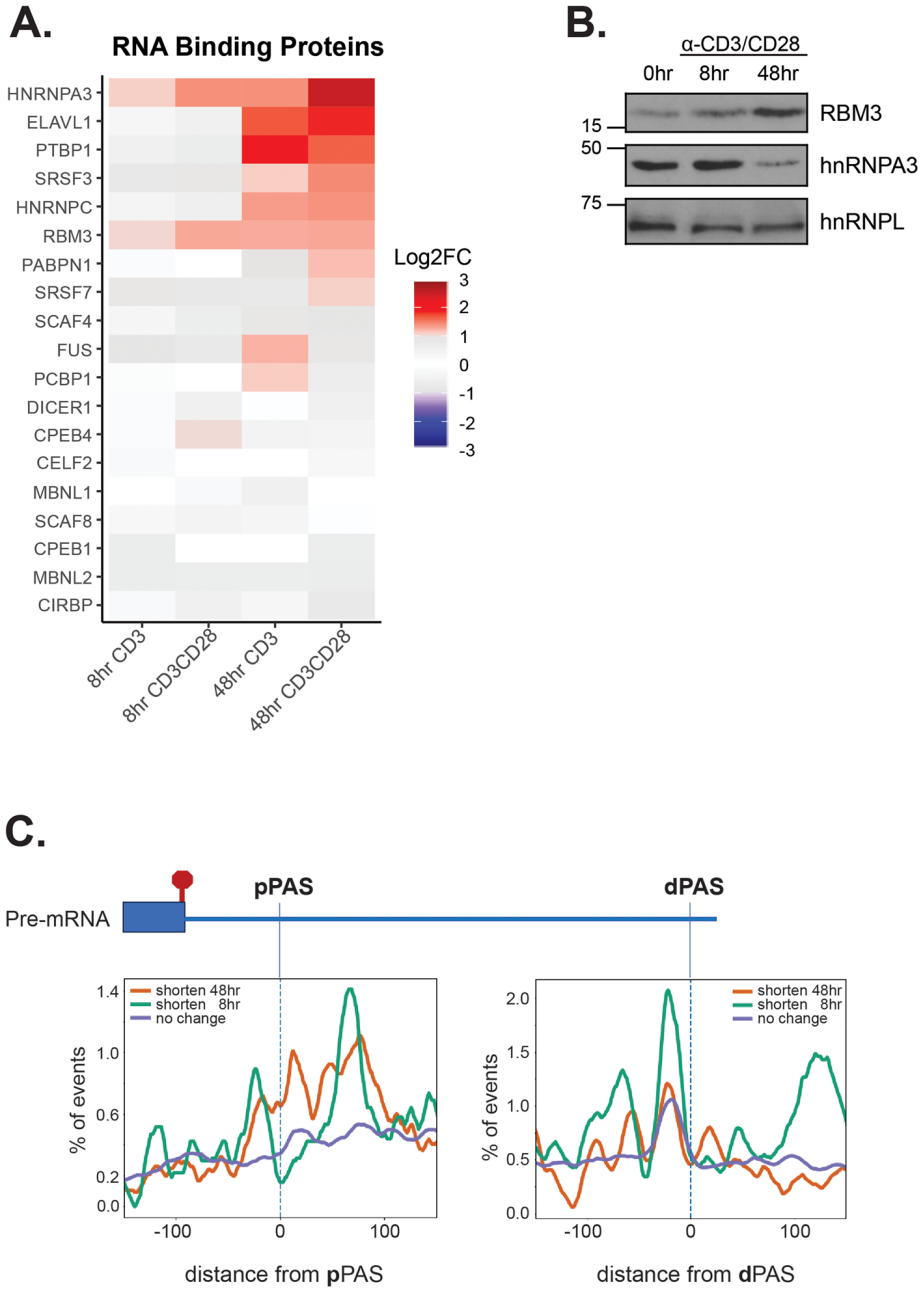
RBM3 increases at 8 h of costimulation of primary human T cells and has its binding motif is enriched around dPAS that are repressed at 8 h. (**A**) Log2 fold changes of proteins that have been implicated in APA regulation. (**B**) Western blot analysis of RBM3 and hnRNP A3 protein following 8 h of CD3/CD28 costimulation of CD4^+^ human T cells. (**C**) Presence of RBM3 binding motifs around pPAS and dPAS elements for events that exhibit shortening at 8 h versus 48 h or show no change.

In contrast to what is observed at 48 h, at 8 h we observe little evidence of cellular proliferation (Supplemental Fig. S6A) and only one of the core polyadenylation factors showed any upregulation in the RNA-Seq analysis (CTSF2, Fig. 4B), but this was not observed by qPCR (Supplemental Fig. S6B) or increased protein (Fig. 4C). Therefore, it is unlikely that regulation of APA at 8 h post-stimulation occurs via increased abundance of the core polyadenylation machinery. To investigate additional mechanism of APA regulation, we assessed expression of genes encoding RNA binding proteins that have been characterized to influence APA changes^5^. Notably, eight of these RBPs are significantly upregulated upon 48 h of CD3/CD28 stimulation with two (HNRNPA3 and RBM3) exhibiting at least a 2-fold change by 8 h of stimulation by RNA-Seq (Fig. 5A). Importantly, for RBM3 this change in mRNA is also mirrored in an increase in protein expression, while for hnRNP A3 protein abundance does not follow mRNA (Fig. 5B). The activation-induced upregulation of RBM3 is further intriguing as the experimentally-determined optimal binding motif for this protein is AAUAUA^37^, which is highly similar to the PAS consensus of AAUAAA. Interestingly, 3′UTRs that exhibit shortening at 8 h reveal enrichment for a AAUAUA motif around the distal PAS site, while no such enrichment was observed around the distal PAS in 3′UTRs that are unchanged or only shorten at 48 h (Fig. 5C, dPAS). By contrast, there is no preferential enrichment of AAUAUA at the promixal PAS site (Fig. 5C, pPAS).

**Figure 6:**
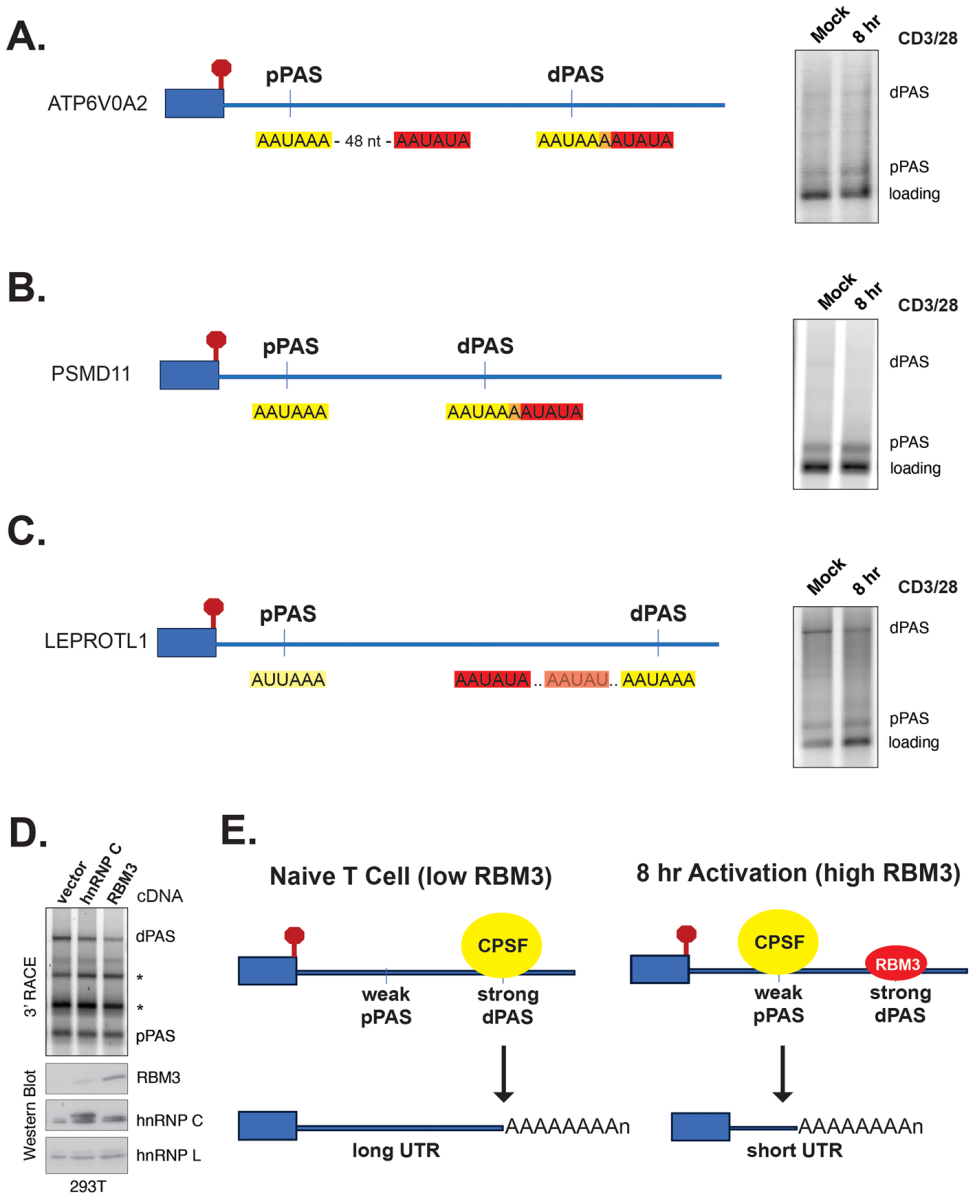
Examples of genes potentially regulated by RBM3 and a model for RBM3-mediated 3′UTR shortening. (**A**–**C**) Left: Schematic of RBM3 motifs adjacent to PAS sequences in ATP6V0A2 (**A**), PSMD11 (**B**) and LEPROTL1 (**C**) genes. Right: 3′RACE showing favoring of pPAS versus dPAS at 8 h of costimulation. (**D**) Top: 3′RACE of LEPROTL1 in 293T cells transfected with cDNA expressing hnRNP C (control) or RBM3 or vector alone. Asterisks mark products observed only in 293T cells. Bottom: Western blot expression of RBM3, hnRNP C and hnRNP L (loading control) in the same cells as top experiment. Expression of hnRNP C causes a small increase in endogenous RBM3. (**E**) Model for 3′UTR shortening by RBM3, showing the suggested competitive binding between RBM3 and the cleavage and polyadenylation machinery (CPSF complex).

We confirmed 3′UTR shortening at 8 h for several genes that have RBM3 motifs overlapping or adjacent to the distal PAS sites (Fig. 6A–C). We note that for most of these genes, including all the ones tested, the distance between the PAS sites is also ~ 1 kb or more. This makes the distal PAS site challenging to detect by 3′RACE, but also perhaps suggest additional mechanistic determinants of regulation. RBM3 has previously been reported to impact APA in mouse fibroblasts^38^. Although we have been unsuccessful in manipulating the levels of RBM3 in human T cells, upon overexpression of RBM3 in 293T we do detect reduced use of the distal PAS site in the one gene that exhibits APA in these cells (Fig. 6D). Taken together, these data suggest a model of regulation in which increased expression of RBM3 early upon T cell activation leads to increased binding of RBM3 to the AAUAUA-containing distal PAS sites, thereby competitively inhibiting association of the polyadenylation machinery to these locations. As a result, association of the polyadenylation machinery with the very upstream PAS sites would be favored as RBM3 levels increase, leading to shortening of the 3′UTRs (Fig. 6E).

## Discussion

It is now well established that 3′UTR identity is highly regulated during many transitions in cell state, including T cell activation, through the regulation of APA^11,12^. However, the mechanisms of regulation and functional consequences remain largely unexplored. Here we present a detailed analysis of the temporal and condition-specific regulation of APA following activation of primary human CD4^+^ T cells. Our data is consistent with previous studies in showing a significant bias in 3′UTRs shortening upon T cell activation^11,12^, but also provide new insight into the functional implications of this regulation and the contribution of CD28 costimulation and RBP expression to controlling APA.

In terms of functional significance, we find enrichment of highly distinct functional categories amongst the genes that undergo 3′UTR shortening at early versus late time points of activation and those that respond to CD28 costimulation, and also observe regulated APA several genes known to have critical function in T cell biology. In addition, while only a minor population of genes exhibit 3′UTR lengthening upon T cell stimulation, these genes are particularly highly enriched for roles in mTOR signaling and regulation, suggesting that this regulatory program while minor in number may have marked impact on cellular function. An additional unexpected finding from our data is that genes that undergo 3′UTR shortening are enriched for association with the protein TIS11B, which targets genes for translation in ER-proximal TIS-granules^21,27^. It will be of interest for future study to determine how TIS11B may contribute to shaping protein and cellular function during T cell activation.

With regards to mechanism of regulation of APA during T cell activation, our data at late time points of costimulation with CD3 and CD28 is consistent with models of 3′UTR shortening upon increased abundance of core polyadenylation factors. In particular, we find dramatic upregulation of CSTF2 and its corresponding protein product CstF64, which is a key enhancer of PAS site usage. Increased expression of CSTF2 is well known to promote proximal site usage of IgM heavy chain transcript within B cells, and there are multiple cases of proliferating cells exhibiting increased expression of CSTF2 and 3′UTR shortening^16,36^. We also observe significant upregulation of the genes and/or proteins comprising the CFI enhancer, namely NUDT21, CPSF6 and CPSF7^35^. At least for NUDT21 and CPSF6, previous studies have found that knockdown of these factors correlates with increased usage of the distal PAS site^35^. Together with the observation in our data and others that proximal PAS sites are typically weak, it follows that increased expression of the core polyadenylation enhancers would preferentially promote proximal PAS usage, thus leading to 3′UTR shortening.

One unresolved question is the mechanism by which CD28 costimulation promotes APA for a subset of genes. One potential mechanism to explain how CD28 costimulation regulates APA changes is through the increased expression of the core polyadenylation factors, CPSF2, and CPSF3, which we observe by RNA-Seq to be enhanced by costimulation with CD28 relative to CD3 alone. The function of CPSF3 is the endonuclease with of the of the complex that recognizes the CA dinucleotide sequence, whereas CPSF2 is another component of the core polyadenylation complex. While these factors do not display sequence specificity, an increase in concentration would be predicted to increase occupancy of weak PAS sites. We also identify several putative regulators of polyadenylation that exhibit enhanced increases in gene expression upon CD28 costimulation, including SRSF3 and SRSF7 and PABPN1. We do not find motifs suggestive of binding of these factors or any other known RBPs enriched around CD28-enhanced APA events; however, these may each regulate a subset of genes such that there is no clear enrichment for any one motif. However, we cannot rule out additional modes of regulation such as via differences in transcription or other indirect mechanisms.

Interestingly, our data do suggest a previously unknown mechanism for APA regulation in T cell via induced expression of RBM3 early upon T cell activation. RBM3 is a stress-induced protein that has been implicated in cytokine expression in innate lymphoid cells^39^, and has neural protective activity^40^. We show here that expression of RBM3 mRNA and protein are markedly enhanced early after T cell activation and this correlates with 3′UTR shortening of genes that have an RBM3 binding motif immediately overlapping the distal PAS site. We also observe a general enrichment of RBM3 binding motifs near distal, but not proximal, PAS sites of genes that exhibit shortening of the 3′UTR within 8 h of T cell activation. Together these data suggest that RBM3 competitively inhibits distal PAS usage to promote 3′UTR shortening of at least a subset of early APA events. Whether this activity of RBM3 contributes to its previously demonstrated role in cytokine expression remains a question for future study.

In sum, we report here a comprehensive analysis of regulation of APA induced upon T cell activation. This work has revealed a broad set of genes that exhibit 3′UTR shortening in a condition and temporal-dependent manner and also identifies a small but important set of genes that experience 3′UTR lengthening in activated T cells. We further provide evidence for the mechanisms of regulation through increased expression of both core polyadenylation factors and enhancers as well as the putative regulatory protein RBM3. Overall, this work provides a deeper understanding of how global APA changes are regulated within activated T cells and highlights the potential biological impact APA on the human immune response.

## Methods

### Isolation and stimulation of primary human CD4^+^ T cells

CD4^+^ T cells were obtained by apheresis from de-identified healthy blood donors after informed consent by the University of Pennsylvania Human Immunology Core. Samples were collected from three donors, Donor 1 (age: 46, sex: male), Donor 2 (age: 26, sex: female), and Donor 3 (age: 32, sex: male). Naïve CD4^+^ T cells were negatively enriched for by utilizing CD45RO microbeads (Miltenyi: 130-046-001). 5 × 10^6^ naïve CD4^+^ T cells were stimulated in complete RPMI supplemented with 10 IU of IL-2 (Miltenyi: 130-097-742), with either soluble 2.5 μg/mL anti-CD28 (BD: 348040), or 2.5 μg/mL bound anti-CD3 (BD: 555336), or with both soluble anti-CD28 and bound anti-CD3. Cells were harvested after 8 h and 48 h of culture. Validation of stimulation is described elsewhere^18^.

#### Access of human cells

Primary human T cells were obtained from the Penn Human Immunology Core (HIC). All methods for cell collection and use were carried out in accordance with IRB guidelines and regulations as approved by the University of Pennsylvania IRB license #703185 to the HIC and IRB #811028 to K.W.L. All cells were obtained from adults with their informed consent.

### RNA-seq data processing

To define the global landscape of APA upon T cell stimulation, we re-analyzed our previously generated RNA-seq data from (CD45R0−) CD4^+^ primary T cells from 3 healthy human donors (GSE135118). Raw sequencing reads had adaptor sequences removed and low-quality base calls were trimmed off using bbduk and resulting reads < 35 nt were discarded. Reads were mapped to the hg38 build of the human genome using STAR. Steady state differential mRNA expression was called using DESeq2. For averaging and normalizing reads, mapped reads at each position across a gene was normalized to reads per million for each samples. Normalized read depth at each position was then averaged across the three replicates and plotted across the bedgraph.

We quantified APA using DaPars where we compared naïve primary T cells from the 3 donors to ex vivo stimulated T cells for 8 h or 48 h with anti-CD3 or co-stimulation with anti-CD3 and anti-CD28. Significant APA shifts were called as those with an absolute change in Percentage Distal Usage Index (dPDUI) between naïve and stimulated conditions greater than 20% with an adjusted p-value less than 0.05. Non-changing events were those with an |dPDUI| < 3% and *p* > 0.05. To focus on late-changing APA events we defined 48 h only events as those that were significantly shortened comparing 48 h stimulated to naïve T cells but defined as non-changing after 8 h of stimulation.

### Flow cytometry proliferation assay

Isolated primary human T cells were incubated with 10 uM CellTrace Violet (C34571, ThermoFisher) for 20 min according to the manufacturer protocol, and then returned to culture dishes and stimulated for the given timepoints described above. About half a million cells per condition were harvested, washed, and resuspended in MACS buffer (DBPS without calcium and magnesium, supplemented with 1% FBS) for analysis by flow cytometry. Forward and side scatter were used to identify live cells, while fluorescence at 450 nM identified amount of CellTrace violet retained. Maximal signal is observed for cells that have not divided. Peaks at lower intensities indicate cell division.

### PAS strength and motif analysis

To compare PAS strength of proximal PAS (pPAS) and distal PAS (dPAS) at 48 h only shortened events we reported the fraction of pPAS and dPAS that had the core PAS hexamer (AAUAAA) or the upstream enhancing element (UGUA) within 100 nucleotides (nt) upstream of the reported cleavage and polyadenylation sites. Our initial analysis found very few DaPars events with proximal sites having these motifs (data not shown), consistent with previous results showing DaPars does not identify/quantify proximal PAS as well as distal PAS^41^. Therefore, we restricted the DaPars event set to those events also found with a second algorithm (PAQR) that more accurately identified proximal sites for the purpose of comparative PAS strength analysis.

For RBM3 motif maps we searched for the putative RBM3 hexamer (AAUAUA) that was not the core PAS hexamer (AAUAAA) around the previously defined DaPars APA events that shortened at 8 or 48 h compared to those that were non-changing. We plotted the fraction of events that contained this hexamer around the pPAS or dPAS by using a sliding window of 10 nucleotides and then smoothing with a running mean of 5 nucleotides.

### Gene ontology enrichment analysis

GO enrichment analysis was performed using the GO biological process compete tool provided by the PANTHER Classification System (http://pantherdb.org). The significant GO enrichment analysis met the threshold of p < 0.05 and an enrichment score > 2.

### qPCR

Total RNA was extracted from cells with Trizol as per the manufacturer instructions and resuspended in water. For cDNA synthesis, reverse transcription was performed with Oligo(dT) and Moloney murine leukaemia virus (M-MLV) reverse transcriptase (Promega) per the manufacturer’s protocol. qPCR was performed with gene specific primers and Sybr Green reagent on the Thermo Applied Biosystems QuantStudio 7 Flex Real-Time PCR. Relative quantification was calculated according to the ΔΔCT method. GAPDH and 18S RNA were used as a housekeeping genes for normalization controls. All qPCR data is from triplicate biologic replicates, each analyzed in technical duplicate. Primer sequences used for qPCR are:

- hnRNPA3-F: GCAATGTGTGCTCGACCACACAA
- hnRNPA3-R: TTCTTCACTGTTAGATGGGCACCAGG
- GAPDH-F: ACCAAATCCGTTGACTCCGACCTT
- GAPDH-R: TCGACAGTCAGCCGCATCTTCTTT
- CSTF2_F: CCCAGTCATGCAGGGAACAGGAATGC
- CSTF2_R: CTCATGATCCTGTGGAGTGACTTGG
- NUDT21_F: AGCCCCTCTACGAGAAGGACAGCTCTG
- NUDT21_R: GCTGCAGCAGTAACACATGGGGTAG
- CPSF3_F: GTCCATGGAGAACAGAATGAAATGGCC
- CPSF3_R: CTCCTCTGAAGTTTAAGGTCACTGCTTC
- CPSF6_F: GTGAACGATCAAGAGAGAGGGACCATAG
- CPSF6_R: CGATGACGATATTCGCGCTCTC
- CPSF7_F: GAAAGGTCACCTAGCCGGTCCCG
- CPSF7_R: CGTTCTCTATCCCGGTGTCTCTCATG

### Transfection of 293T cells

A plasmid with the RBM3 cDNA driven from the CMV promoter was purchased from Origene (cat # SC322227) and transfected into 293T cells using Lipofectamine 2000 (Thermo Fisher) according to the manufacturer’s protocol. As a control, an expression plasmid for hnRNP C described previously^42^ was transfected in parallel by the same method. 24 h after transfection, protein and RNA were harvested from cells for 3′RACE and Western blotting, respectively.

### Western blotting

10 µg of total protein lysates were loaded into 10% 37.5:1 bis-acrylamide SDS-PAGE gels. SDS-PAGE gels were transferred onto PDVF membranes and targeted proteins were visualized with a chemiluminescence system and subsequent imaging with an X-ray developer. Antibodies used to detect protein expression levels are as follows: CSTF2 (abcam: ab72297), CPSF3 (abcam: ab72299), CPSF6 (Santa Cruz: sc-100692), CPSF7 (Bethyl Laboratories: A301-360A), NUDT21 (abcam: ab183660), hnRNP L (abcam: ab6106), hnRNP A3 (abcam: ab78300), RBM3 (Proteintech: 14363-1-AP).

### Validation of APA changes using 3′RACE

APA events were validated using a PCR based assay, 3′RACE. With RNA harvested from cells, 3′RACE was done using the SMARTer^®^ RACE 5′/3′ Kit (634859) according to the manufacture instructions. 3′RACE PCR product was analyzed with ethidium bromide stained agarose gels. Primers used to amplify 3′UTR products are as follows:

- Loading control/SDHA: CGAGGCTGACTGTGCCACCGTCC
- NFATC2IP: GCAGCCTCTGTGACTTGGAGATGTCCC
- Bid: GGAGCAGCTGCTGCAGGCCTACCCTAG
- cFLIP (primer 1): AGCAGCTCTAGCTGCCATCTTGAACC
- cFLIP (primer 2): CTTGGAAAGTATGCTGTAGCTCAAGTGC
- ATP6V0A2: GTTGTCTGATGTCCTGTGGGCCATGC
- PSMD11: GGACCAGGGGGAGGGTGTCCTG
- LEPROTL1: CACCTATTCCATACTGCATAGCAAGAGG

### Supplementary Information

Supplementary Figures.

## Data Availability

All data generated or analyzed during this study are included in this published article and its supplementary information files.
