# Supplementary material for: Alternative 3′UTR expression induced by T cell activation is regulated in a temporal and signal dependent manner
Source: Sci Rep. 2024 May 14;14:10987. doi: 10.1038/s41598-024-61951-1 (PMC11094061; doi:10.1038/s41598-024-61951-1)
Supplement: Supplementary file 1 — Supplementary Figures. [file 41598_2024_61951_MOESM1_ESM.pdf]

Supplemental Fig S1

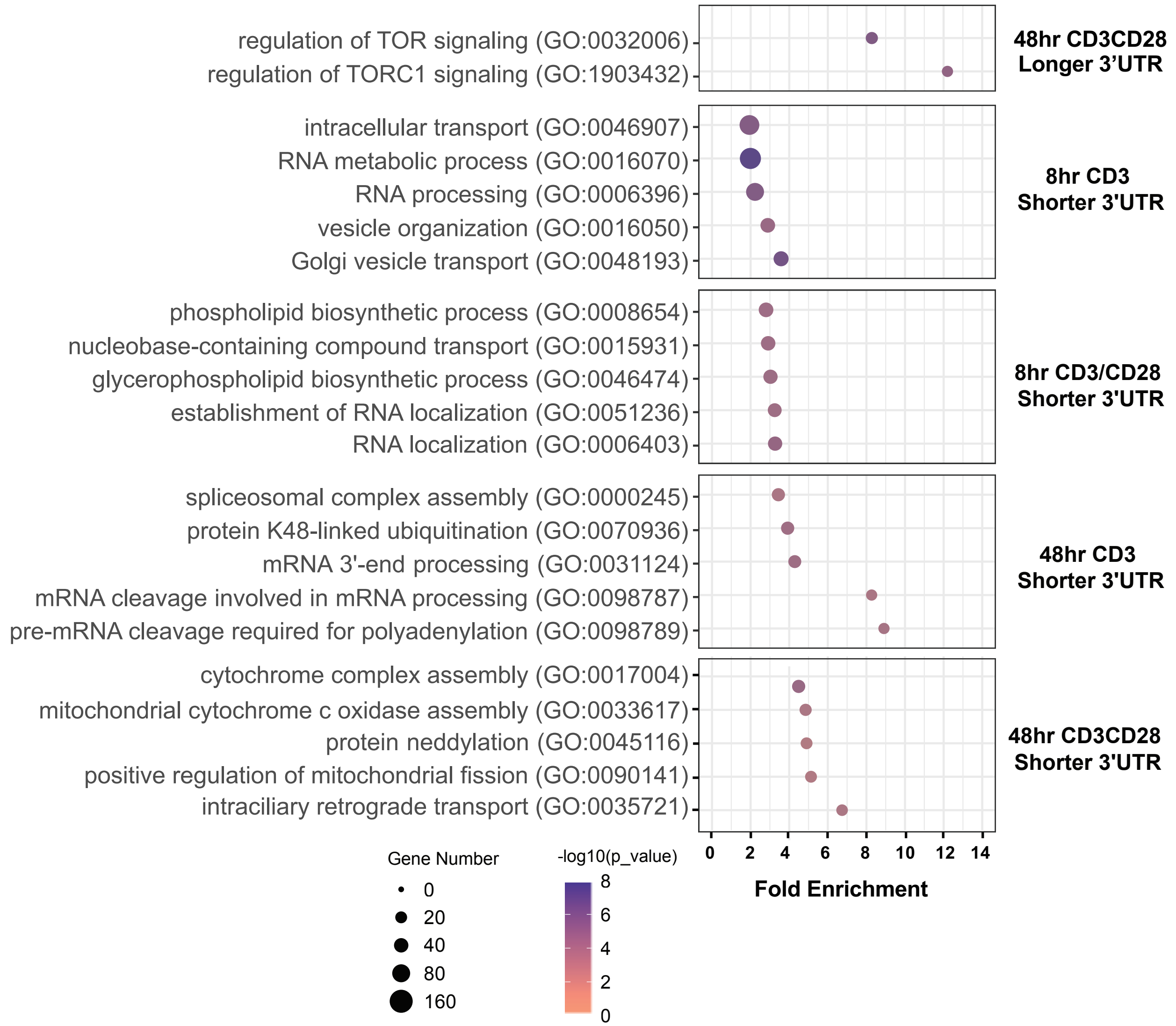

Supplemental Fig S2

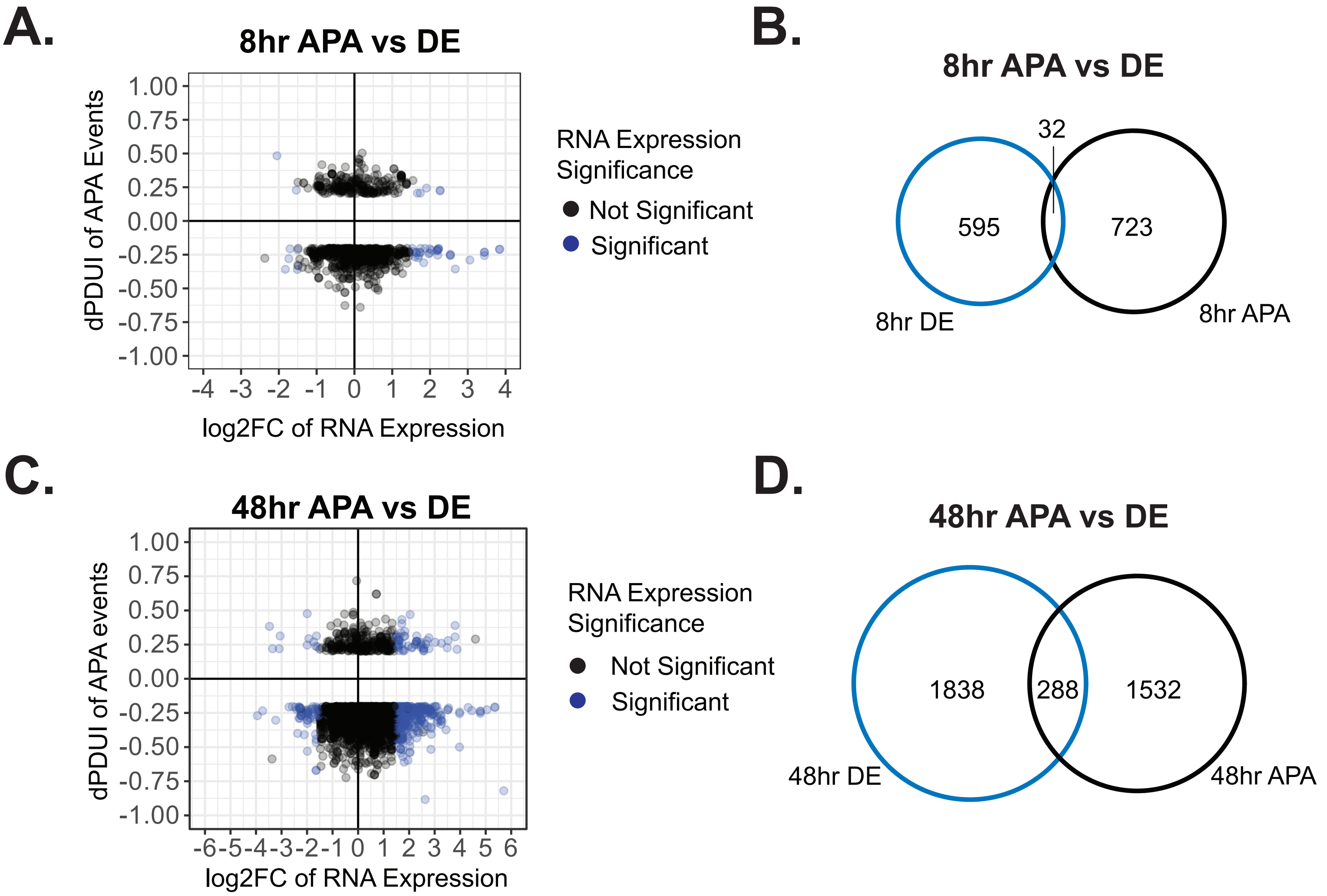

Supplemental Fig S3

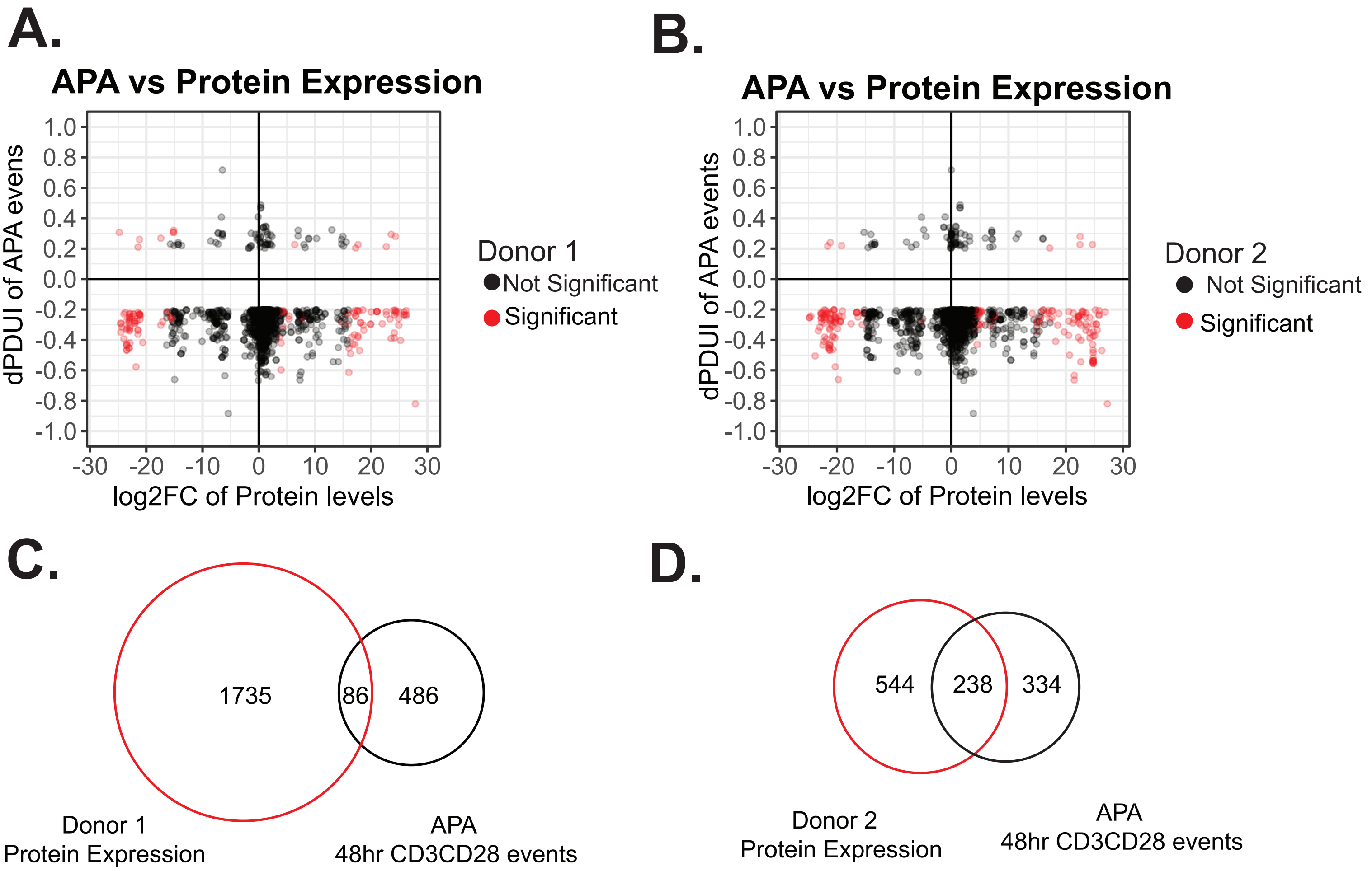

Supplemental Fig S4

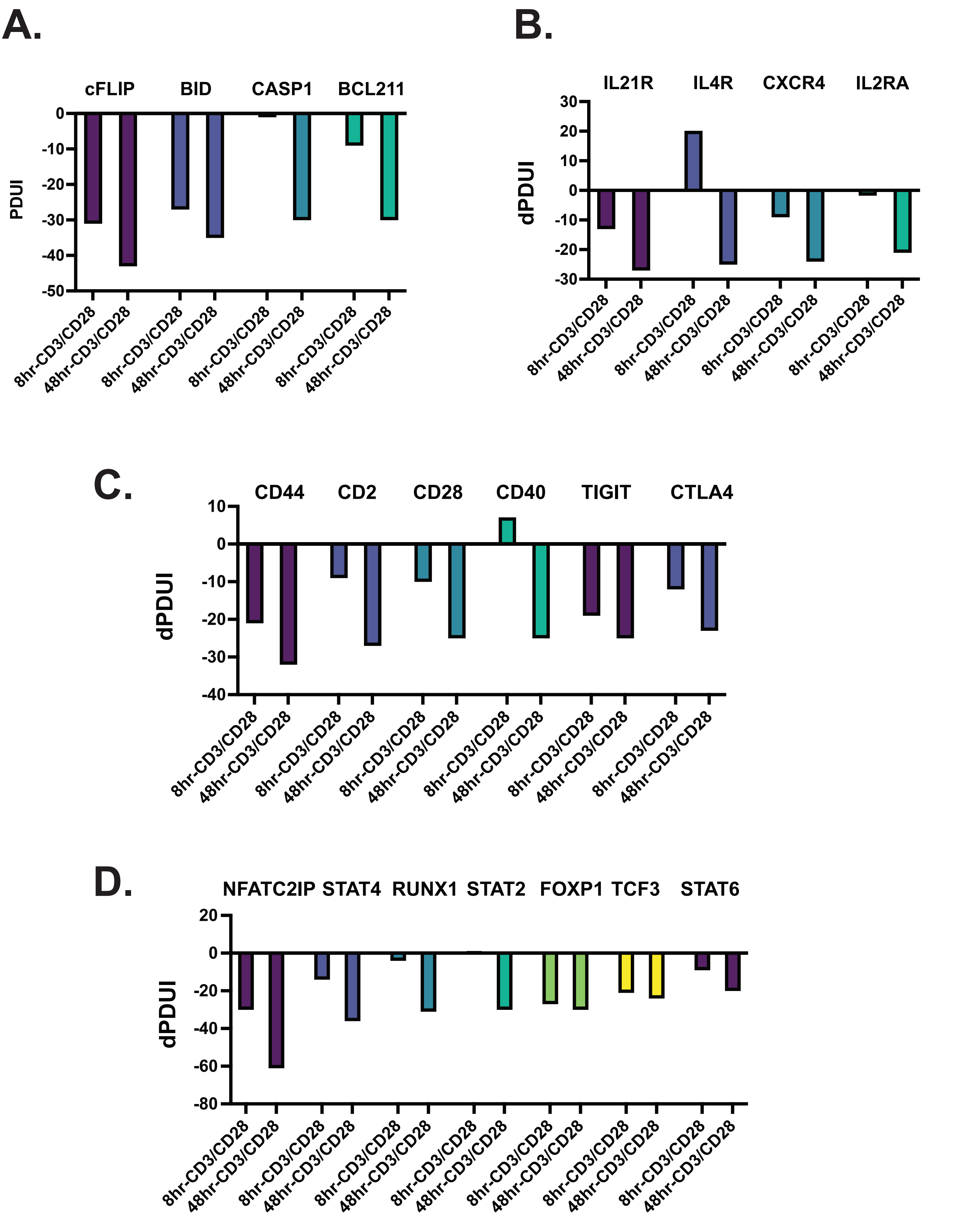

Supplemental Fig S5

A.

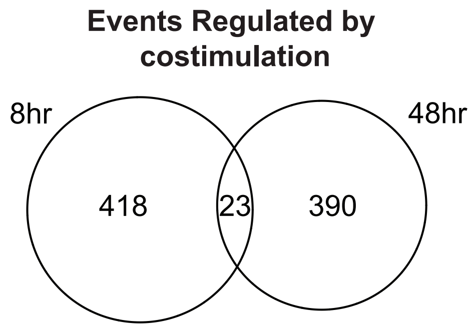

B.

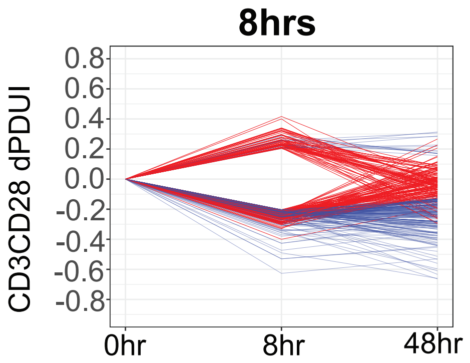

C.

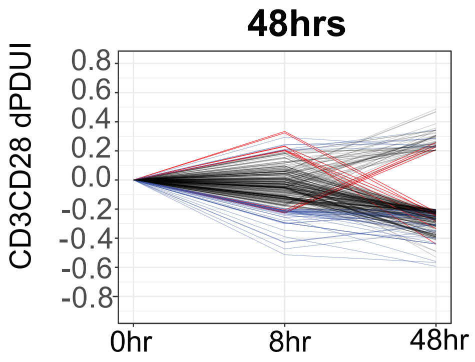

	Category	# of Events
(red)	Early/Transient Changes	209
(blue)	Early/Sustained Changes	170
(black)	Late Changes	0

	Category	# of Events
(red)	Early/Transient Changes	12
(blue)	Early/Sustained Changes	44
(black)	Late Changes	285

Supplemental Fig S6

A.

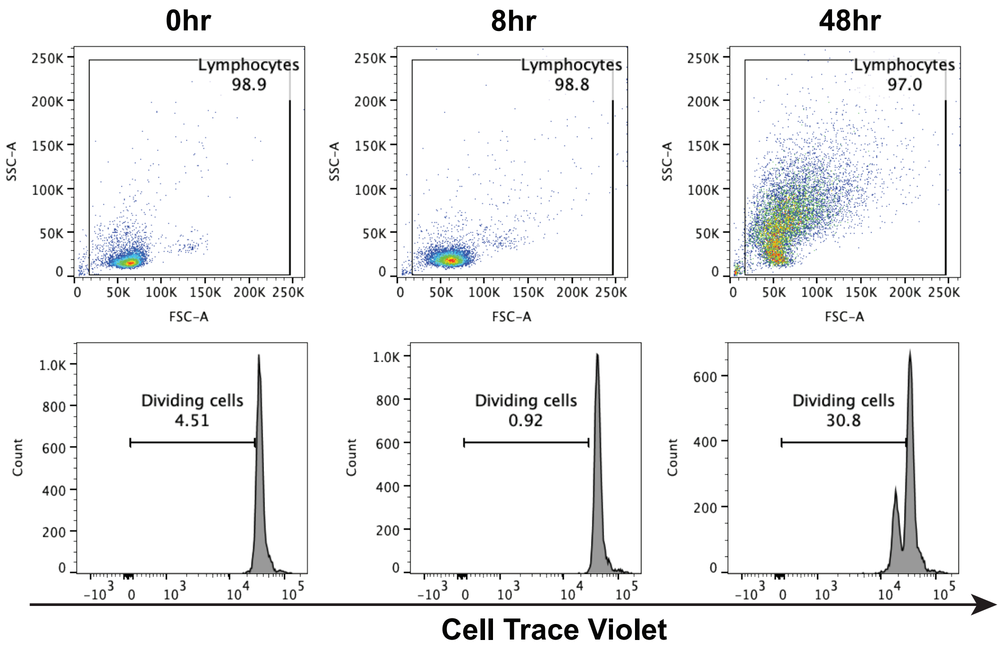

B.

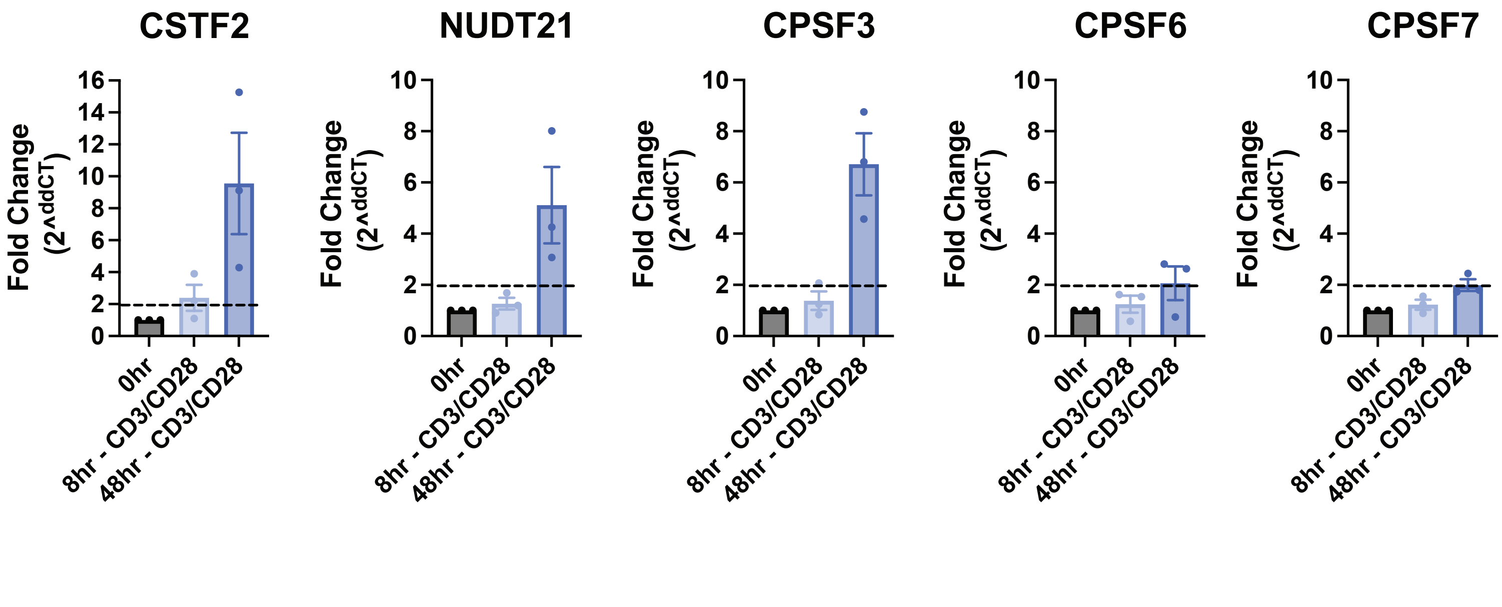

Supplemental Fig S7

Full uncropped gels for Figure 2

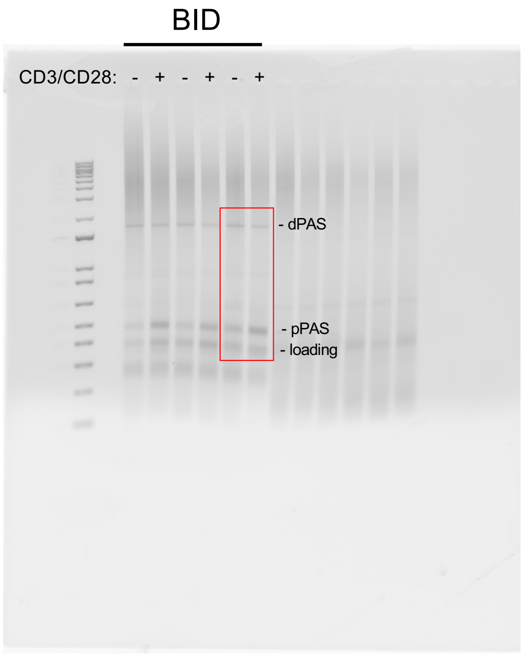

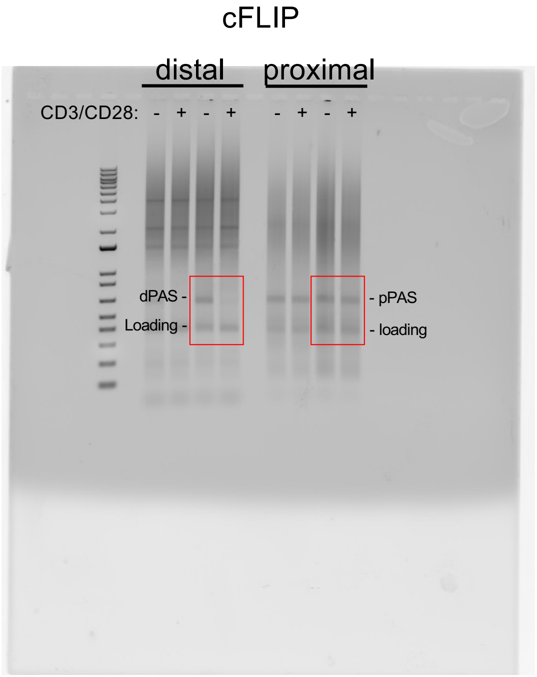

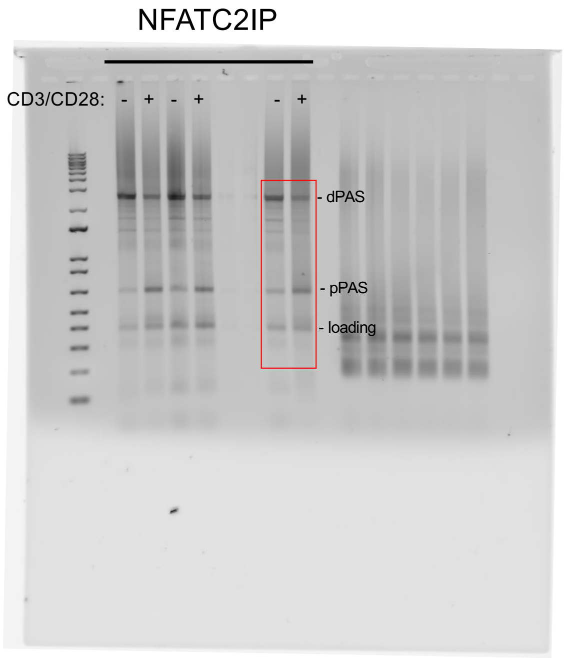

Supplemental Fig S7 (cont)

Full uncropped gels for Figure 4

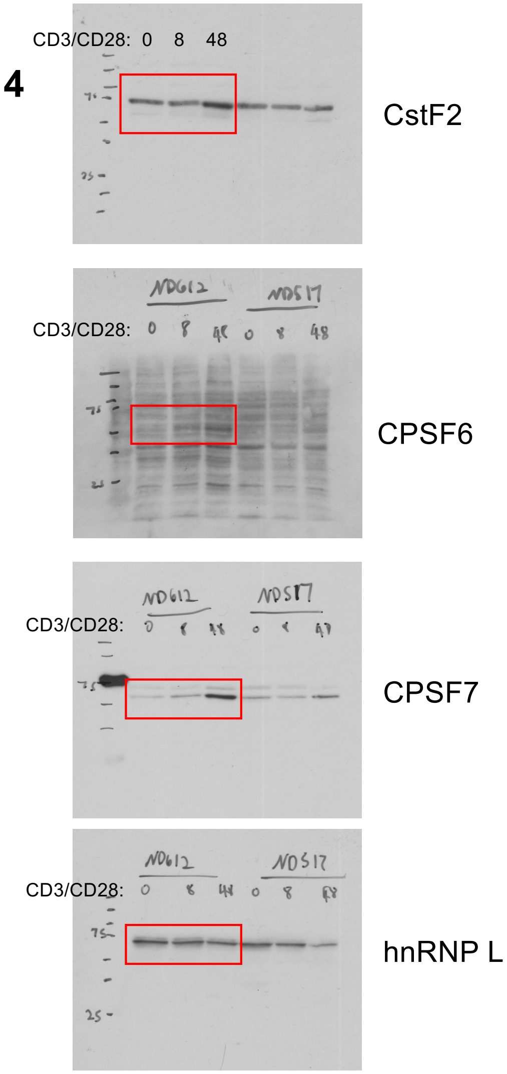

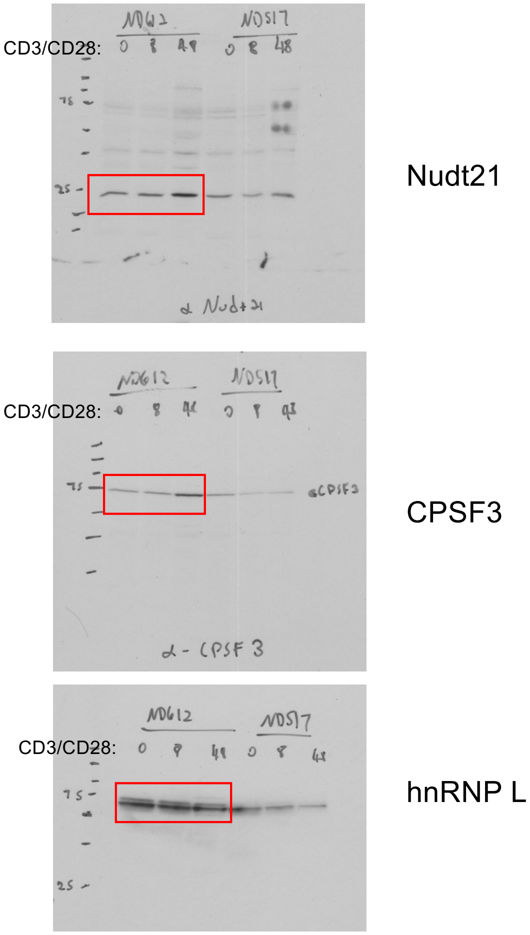

Supplemental Fig S7 (cont)

Full uncropped gels for Figure 5

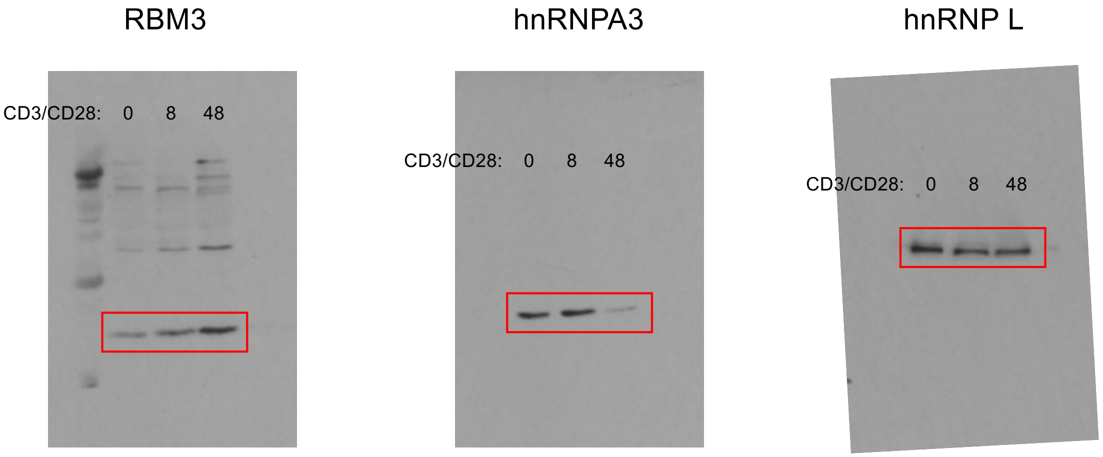

Supplemental Fig S7 (cont)

Full uncropped gels for Figure 6

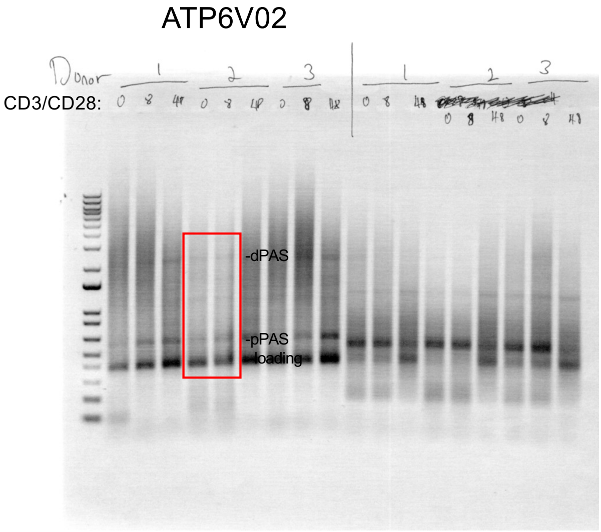

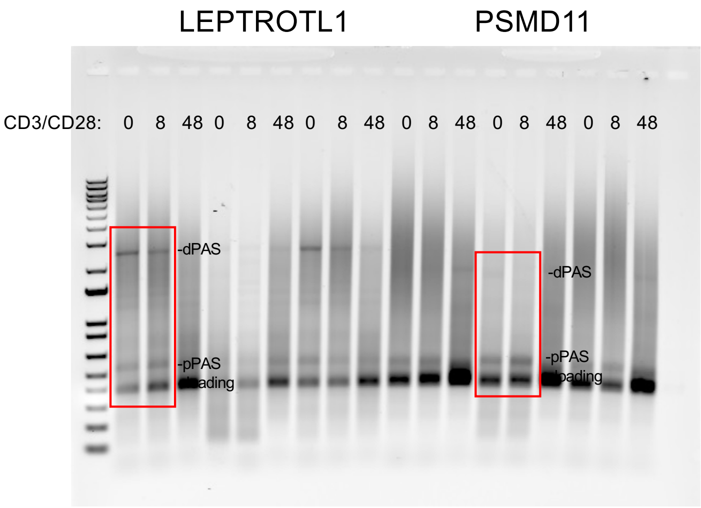

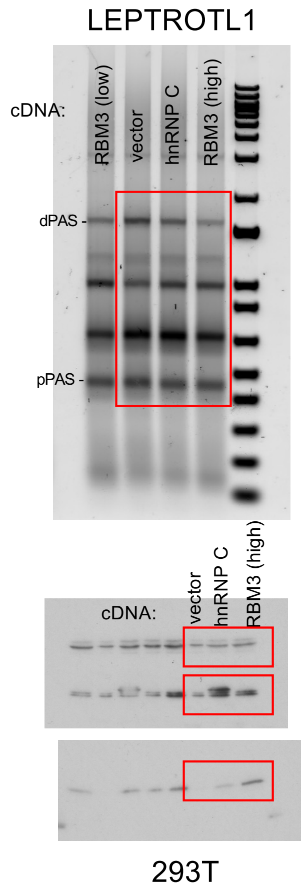

Supplemental Figure Legends

Supplemental Figure S1: Gene ontology analysis of shortened and lengthened 3'UTR upon T cell activation. Gene ontology analysis for significant ($p < .05$) biological enrichment of significant APA events. APA events are grouped by shortened or lengthened 3'UTRs that are exhibited upon T cell stimulation.

Supplemental Figure S2: APA does not correlate globally with gene expression. (A) Dot plot of genes selected based on significant APA changes induced upon 8hr-CD3/CD28 stimulation. Dots highlighted in blue are genes significantly (> 1.5 log2FC, < 0.5 p-value) coregulated by differential RNA upon 8hr-CD3/CD28 induction. (B) Overlap of genes coregulated by significant APA and RNA expression changes at 8 hours. (C,D) Same as panels A and B but for genes coregulated by APA and RNA expression changes at 48 hours of CD3/CD28 stimulation.

Supplemental Figure S3: APA does not correlate globally with protein expression. Correlation plots of APA with publicly available proteomic data[45]. (A) Dot plot of genes selected based on APA significance induced upon 48hr-CD3/CD28 stimulation. Dots highlighted in red are genes significantly (> 2 log2FC, < 0.5 p-value) coregulated by differential protein expression upon 72hr-CD3/CD28 induction of one CD4+ T cells isolated from one donor, as quantified by published mass spectrometry analysis[45]. (B) Same as above, however, the protein expression analysis was replicated with an additional donor. (C,D) Overlap of genes coregulated by significant APA and protein expression changes.

Supplemental Figure S4: Details of APA of immune-relevant genes. dPDUI at 8 and 48 hours, as quantified by DaPars, of genes related to apoptosis (A), cytokine receptors (B), cell surface markers (C) and key transcription factors (D). Colors of the bars are arbitrary and solely for the purpose to help distinguish data for one gene from the neighboring data.

Supplemental Figure S5: Temporal characterization APA events regulated by CD28 costimulation. (A) Overlap of significant APA events regulated by CD28 costimulation between 8 and 48 hours of stimulation. (B-C) Analysis of temporal trends between 8 and 48 hours of T cell stimulation of APA events regulated by CD28 costimulation. Events labeled as “Early/Transient Changes” have a significant change at 8 hours but a decrease in dPSI of >2 by 48 hours, while “Early Sustained Changes” were significant at 8 hours and showed no decrease of more than 2-fold by 48 hours. Lastly, remaining events were labeled as “Late Changes”.

Supplemental Figure S6: Proliferation and increased expression of polyadenylation machinery is observed at 48, but not 8, hours after costimulation of primary CD4+ T cells (A) Proliferation assay by flow cytometry using Cell Trace Violet to observe cell division. (B) qPCR validation of mRNA expression for genes encoding key components of the polyadenylation machinery.

Supplemental Figure S7: Full uncropped gels. Uncropped gels for 3’RACE and Western blot images in Figures 2,4,5 and 6 are provided.
